# Development and temporal exploratory evaluation of a multi-criteria decision analysis framework for Rh antigen-matched red blood cell donor prioritization

**DOI:** 10.3389/fmed.2026.1962032

**Published:** 2026-10-16

**Authors:** Jun Zhang, Wei Xue, Xiaolu Xie, Xinghua Li

**Affiliations:** 1 Department of Blood Transfusion, Affiliated Hospital of Yangzhou University, Yangzhou, Jiangsu, China; 2 Department of Blood Transfusion, The First Affiliated Hospital of Kangda College of Nanjing Medical University (The First People's Hospital of Lianyungang), Lianyungang, Jiangsu, China

**Keywords:** decision support, donor prioritization, irregular antibodies, multi-criteria decision analysis, red blood cell transfusion, rh antigen matching, TOPSIS

## Abstract

**Background:**

Rh antigen matching is an established strategy for reducing red blood cell (RBC) alloimmunization after transfusion. However, routine pretransfusion testing primarily determines whether a donor unit is acceptable on the basis of ABO/RhD compatibility, antibody-screening results, and crossmatch compatibility. For recipients with a negative pretransfusion antibody screen, selection of the optimal unit among multiple acceptable RBC candidates remains largely dependent on empirical judgment and inventory availability.

**Methods:**

An MCDA framework was constructed using RhD-positive RBC transfusion data, incorporating RhC, c, E, and e antigen matching characteristics together with inventory-related considerations. Expert preferences were quantified using the analytic hierarchy process (AHP), and donor antigen-profile options were ranked using the technique for order preference by similarity to ideal solution (TOPSIS). The expert panel included seven professionals with backgrounds in transfusion medicine or clinical laboratory practice. The AHP-derived weights assigned to Rh antigen criteria were 0.5528 for c, 0.2687 for E, 0.1202 for e, and 0.0583 for C. At the unit level, high match was defined as Rank 1, or Rank 2 with an Rh antigen matching score of at least 9 and an integrated priority score within 10% of the highest-ranked option. Units not meeting these criteria were considered low match. For patients receiving multiple RBC units, high-match status required all transfused units to meet the unit-level high-match criteria; otherwise, the patient was classified as low match. Patients were categorized according to the concordance between the actual transfused donor RBC unit and the MCDA-derived donor-unit priority ranking, and the association between matching status and newly detected irregular antibody positivity within 6 months was assessed.

**Results:**

The MCDA framework generated structured donor priority rankings across different recipient Rh phenotypes. The ranking order remained consistent under alternative weight perturbation scenarios, supporting the stability of the proposed prioritization approach. The temporal exploratory cohort included 313 patients, with 156 classified into the high-match group and 157 into the low-match group. The incidence of newly detected irregular antibodies within 6 months was 3.2% (5/156) in the high-match group and 11.5% (18/157) in the low-match group, representing an absolute difference of 8.3 percentage points. Firth-corrected logistic regression analysis showed that high-match status was associated with a lower likelihood of newly detected irregular antibodies compared with low-match status [odds ratio [OR], 0.29; 95% confidence interval [CI], 0.10–0.82]. Sensitivity analyses yielded consistent results.

**Conclusion:**

We developed an MCDA-based Rh antigen-matched RBC donor prioritization framework that enables structured and interpretable ranking among multiple clinically acceptable transfusion options. In this temporal exploratory evaluation, greater concordance between actual transfusion practice and MCDA-based ranking was associated with a lower incidence of newly detected irregular antibodies. The proposed framework is intended to complement, rather than replace, conventional immunohematologic testing. It may provide additional decision support when multiple compatible RBC units are available. Further multicenter prospective studies are warranted to determine its feasibility, clinical utility, and impact on transfusion practice.

## 1 Introduction

With the evolution of precision transfusion medicine, red blood cell (RBC) antigen matching has expanded beyond conventional ABO and RhD compatibility to include clinically relevant non-D Rh antigens. The Rh blood group system is highly immunogenic, and mismatches involving C, c, E, and e antigens may induce alloimmune responses, leading to irregular antibody formation, challenges in compatibility testing, delayed hemolytic transfusion reactions, and increased risks during subsequent transfusion episodes，and, in females of childbearing potential, hemolytic disease of the fetus and newborn in future pregnancies**.** Accordingly, extending Rh antigen matching beyond ABO and RhD compatibility has become an important approach for improving transfusion safety and supporting recipient-phenotype–specific transfusion strategies (1–4).

Extended RBC antigen matching is currently recommended mainly for patients with a high risk of alloimmunization, including those receiving chronic transfusion therapy, individuals with previous pregnancy or transfusion exposure, and patients with known irregular antibodies. Previous studies have shown that matching for RhD, C, E, and Kell (K) antigens can reduce alloantibody formation in chronically transfused populations, while advances in Rh genotyping have improved the accuracy of antigen matching in patients with complex transfusion requirements (5, 6). Here, compatibility assessment refers to determining whether a donor unit meets ABO/RhD requirements, avoids antigens corresponding to known clinically significant antibodies when present, and is crossmatch-compatible; it does not imply that all recipients have pre-existing Rh antibodies. Conventional approaches generally determine whether a specific RBC unit is acceptable for transfusion rather than how to prioritize among multiple acceptable options (7, 8). In routine practice, several ABO/RhD-compatible and crossmatch-compatible RBC units may be available simultaneously, and unit selection often depends on local practice patterns, clinical experience, and inventory conditions. A systematic and reproducible approach for prioritizing these acceptable donor options is therefore needed.

When multiple compatible RBC units are available, conventional categories such as “identical,” “compatible,” or “alternative” provide limited guidance for selecting the optimal option among clinically acceptable choices. Further development of precision transfusion strategies requires decision frameworks that consider not only compatibility, but also antigen exposure, immunological relevance, and practical constraints in transfusion services (9).

The concept of patient blood management (PBM) has further broadened transfusion decision-making from a focus on transfusion safety alone toward a more comprehensive consideration of immunological risk, resource utilization, and inventory management (10, 11). Nevertheless, current research on RBC antigen matching has largely concentrated on specific high-risk populations or individual matching strategies. A quantitative decision-support approach for ranking multiple donor units that already satisfy transfusion requirements has not yet been established.

Multi-criteria decision analysis (MCDA) provides a structured approach for integrating multiple criteria in complex decision-making and has been increasingly applied in healthcare resource allocation, clinical decision support, and health policy research (12, 13). Within the MCDA framework, the analytic hierarchy process (AHP) allows expert judgments and multidimensional evidence to be converted into relative weights, whereas the technique for order preference by similarity to ideal solution (TOPSIS) enables comparative ranking of candidate options based on predefined criteria (14, 15). Previous applications of MCDA in healthcare have demonstrated its potential for addressing complex decisions involving multiple competing considerations (16–18).

In this study, we used recipient–donor-unit transfusion pair data from the Yangzhou region to characterize RhC, c, E, and e antigen distributions and establish a relative weighting system for antigen-matching criteria.

An AHP-based weighting approach was combined with TOPSIS to develop an MCDA framework for prioritizing Rh antigen-matched RBC donor units. In addition, donor RBC storage duration was incorporated as an auxiliary factor to reflect real-world inventory constraints. Rather than replacing conventional immunohematological testing, this framework was designed to provide an additional decision-support tool for selecting among multiple clinically acceptable RBC units and to explore a more transparent and quantitative approach for transfusion prioritization.

## 2 Materials and methods

### 2.1 Study population

This study was conducted using transfusion data from the eastern and western campuses of the Affiliated Hospital of Yangzhou University. An MCDA-based framework was developed to prioritize Rh antigen-matched RBC donor units and was subsequently evaluated through a temporal exploratory analysis in a real-world transfusion setting.

During the framework development phase, recipient pretransfusion blood samples collected between January 13, 2023, and August 21, 2024, were linked with samples from the corresponding transfused donor RBC units obtained from the Yangzhou Central Blood Station. Each linked recipient sample and donor-unit sample was defined as one recipient–donor-unit transfusion pair. A total of 11,918 recipient–donor-unit transfusion pairs from 3,455 transfusion recipients were included.These data were used to characterize RhC, c, E, and e antigen distributions and to construct the MCDA-based donor prioritization framework by integrating immunological evidence with practical transfusion considerations.

Among the 3,455 recipients, 3,444 were RhD-positive. RhD-negative recipients were not included because their donor-unit allocation follows a distinct strategy centered on the provision of RhD-negative RBCs, whereas the present framework was designed to prioritize non-D Rh antigen matching among eligible RhD-positive RBC units. The distribution of RhC/c/E/e antigen antigen profiles in RhD-positive recipients is shown in Table 1 (Panel B).

**Table 1:** Distribution of ABO blood groups, RhD status, and serologically detected Rh antigen profiles in the study population.

Panel A. Distribution of ABO blood groups and RhD status (*n* = 3455)		
Characteristic	Patients, n	Percentage (%)
ABO blood group		
A	1144	33.11
B	876	25.35
O	1136	32.88
AB	299	8.65
RhD status		
RhD-positive	3444	99.68
RhD-negative	11	0.32

Panel B. Distribution of serologically detected Rh antigen profiles among RhD-positive transfusion recipients (*n* = 3444)		
Detected Rh antigen profile	Patients, n	Percentage (%)
CDe	1480	42.97
CcDEe	1247	36.21
CcDe	339	9.84
cDE	232	6.74
cDEe	107	3.11
cDe	12	0.35
CcDE	8	0.23
CDEe	19	0.55
Total	3444	100.00

Data are presented as *n* (%). Rh antigen profiles were defined by serologic detection of C, c, E, and e antigens. The profile labels indicate antigen expression only and do not imply genotype or antigen homozygosity. Panel B includes only RhD-positive transfusion recipients.

After framework development, a temporal exploratory evaluation cohort was established to assess the applicability and potential stratification value of the MCDA framework in routine transfusion practice. Consecutive adult patients receiving RhD-positive leukocyte-reduced suspended RBC transfusions between September 2024 and June 2025 were included.

The inclusion criteria were:

(1) receipt of RhD-positive leukocyte-reduced suspended RBC transfusions;
(2) available pre-transfusion ABO, RhD, and RhC/c/E/e antigen phenotype results;
(3) negative pre-transfusion irregular antibody screening;
(4) available follow-up information, including post-transfusion irregular antibody screening within 6 months, transfusion reaction records, and subsequent compatibility testing results.

The exclusion criteria were:

(1) previously identified irregular antibodies or positive antibody screening before transfusion;
(2) autoimmune hemolytic anemia or other conditions potentially affecting antibody detection;
(3) incomplete transfusion records, antibody screening results, or follow-up data;
(4) emergency transfusion scenarios in which Rh antigen matching assessment could not be completed.

Patients in the temporal exploratory cohort were not prospectively assigned RBC units according to MCDA recommendations. Instead, the study retrospectively assessed the concordance between actual transfused units and MCDA-derived donor priority rankings under routine transfusion conditions. For each transfusion episode, Rh antigen matching scores, storage-duration scores, and integrated priority scores were calculated based on recipient Rh phenotype, transfused RBC antigen profiles, and available inventory information. Associations between ranking concordance and post-transfusion immune-related outcomes were then explored.

The purpose of this analysis was to evaluate the applicability and interpretability of the MCDA framework as a donor prioritization tool rather than to assess the effectiveness of MCDA-guided transfusion allocation. The framework was evaluated as a decision-support approach for ranking clinically acceptable RBC units, and the observed associations with immune-related outcomes were considered exploratory.

### 2.2 Development of an MCDA-based framework for Rh antigen-matched donor prioritization

An MCDA-based framework was developed to rank multiple donor options that met routine transfusion requirements. For terminological consistency, a “donor antigen-profile option” refers to a distinct theoretical RhC/c/E/e antigen combination used in the development and illustration of the MCDA model. An “eligible donor RBC unit” refers to an individual RBC unit in the available inventory that satisfied routine ABO/RhD compatibility and crossmatch requirements. “Donor-unit priority ranking” refers to the ordered ranking of eligible donor RBC units according to their final integrated priority scores. MCDA allows several criteria to be considered within a single decision structure and has been used in healthcare resource allocation and clinical decision support (13, 14).

In this study, MCDA served as the overall decision framework: eligible donor units were treated as candidate alternatives, and avoidance of mismatched RhC, c, E, and e antigen exposure was treated as the set of decision criteria. AHP was used to convert expert comparisons of these criteria into relative weights, whereas TOPSIS used the resulting weights to rank the eligible donor options.

The framework incorporated RhC, c, E, and e antigen matching together with inventory-related considerations. Relative antigen weights were derived using the analytic hierarchy process (AHP), and donor antigen-profile options were ranked using the technique for order preference by similarity to ideal solution (TOPSIS) (14, 15).

In this study, recipient-specific information consisted of the recipient's serologically determined RhC, c, E, and e phenotype. For each recipient, eligible donor units were evaluated according to their antigen profiles relative to the recipient phenotype, including whether a donor unit carried an Rh antigen absent from the recipient. Donor-unit storage duration was included only as an auxiliary inventory-related factor. Age, sex, diagnosis, previous transfusion history, immune status, and other clinical characteristics were not incorporated into the ranking calculation. Accordingly, the framework represents recipient-phenotype–specific donor prioritization rather than comprehensive individualized risk prediction. Unlike conventional Rh antigen matching, which generally classifies donor units as identical, compatible, or alternative, the proposed framework generates a weighted priority ranking among multiple eligible units. Unlike inventory-based allocation, it retains Rh antigen matching as the primary decision component and uses storage duration only as a secondary adjustment among otherwise similarly matched units.

(1) Decision Criteria

RhC, c, E, and e antigen matching were selected as the main decision criteria. Their inclusion was based on published evidence on Rh antigen immunogenicity, regional antigen distributions, and routine transfusion practice.

Published immunogenicity data summarized in Supplementary Table S1 informed expert judgment but were not entered directly as MCDA weights.

Final weights were obtained from the AHP pairwise comparison matrix.

(2) AHP Weighting

AHP was used to quantify expert preferences and determine the relative contribution of each Rh antigen criterion to donor prioritization. The resulting weights reflected decision preferences within the ranking framework. They were not intended to estimate the intrinsic immunogenicity of individual antigens or the alloimmunization risk of individual patients.

The expert panel comprised seven professionals: five specialists in transfusion medicine and two in clinical laboratory medicine. Each expert completed the pairwise comparisons independently.

Before scoring, the panel received standardized instructions covering the purpose of the framework, the definition of each criterion, and the Saaty 1–9 scale. Experts then compared the relative importance of RhC, c, E, and e antigen matching on the basis of published evidence and clinical experience.

Individual judgment matrices were aggregated element-wise using the geometric mean. For each pair of criteria, experts judged which antigen-matching criterion was more important and the strength of that preference. The Saaty scale ranges from 1, indicating equal importance, to 9, indicating extreme importance; reciprocal values indicate the corresponding preference in the opposite direction. The principal eigenvector represents the set of relative priorities that best summarizes all pairwise comparisons in the aggregated matrix. After normalization so that the values summed to 1, its components were used as the final criterion weights.

The final aggregated reciprocal matrix used to derive the criterion weights is presented in Supplementary Table S2A. Criterion weights were calculated from the principal right eigenvector of this matrix, and the resulting weights were used in all subsequent TOPSIS analyses. The maximum eigenvalue (*λ*max) was 4.0076, the consistency index (CI) was 0.0025, and the consistency ratio (CR) was 0.0028. A CR below 0.10 was considered acceptable.

The final aggregated pairwise comparison matrix, derived weights, consistency evaluation, and expert panel characteristics are detailed in Supplementary Tables S2A–E. Because expert judgment is inherently uncertain, the AHP-derived weights were used only for donor ranking and not for individual risk prediction.

(3) TOPSIS Ranking

TOPSIS was applied after the AHP weights had been derived. In practical terms, TOPSIS compares each eligible donor option with two theoretical reference options: a positive ideal option with the most favorable values across all criteria and a negative ideal option with the least favorable values. After normalization and application of the AHP weights, the distance of each donor option from these two reference points was calculated. The TOPSIS closeness coefficient was calculated as $Ci=Di-/(Di++Di-)C_{-}i=D_{-}i^{\wedge }-/(D_{-}i^{\wedge }++D_{-}i^{\wedge \_})$, where $Di+D_{-}i^{\wedge }+$ and $Di-D_{-}i^{\wedge \_}$ denote the distances from the positive and negative ideal options, respectively. A higher coefficient indicates that the donor option is closer to the ideal solution and therefore receives a higher priority ranking. The method ranks each option according to its relative distance from the positive and negative ideal solutions (15, 16).

For each recipient Rh phenotype, a decision matrix was constructed from the antigen profiles of eligible donor units and weighted using the AHP-derived values. A higher closeness coefficient indicated a higher priority within the MCDA framework. The TOPSIS closeness coefficient was used to establish the antigen-based priority order of donor antigen-profile options**.** This TOPSIS-derived order was subsequently represented by a prespecified Rh antigen matching score on a 0–10 scale to facilitate clinical interpretation; the Rh antigen matching score was not an independent ranking model.

Representative donor antigen-profile rankings are shown in Table 2. Reference TOPSIS priority scores and related antigen-exposure indicators for the donor antigen-profile options used in the decision framework are provided in Supplementary Table S3.

**Table 2:** Representative donor antigen-profile priority rankings for selected recipient Rh antigen-expression profiles.

Recipient Rh antigen-expression profile	Rank 1 donor antigen-profile option	Rank 2 donor antigen-profile option	Rank 3 donor antigen-profile option	Priority interpretation
CDe	CDe	CcDe	CcDEe	Same phenotype preferred
CcDe	CcDe	CDe	cDe	High matching priority
cDe	cDe	CcDe	CDe	Antigen-conserving priority
CDEe	CDEe	CcDEe	CDe	High matching priority
CcDEe	CcDEe	CDEe	cDEe	Preferred compatible option
cDEe	cDEe	CcDEe	cDe	Reduced additional antigen exposure
CcDE	CcDE	CcDEe	cDE	Priority based on antigen-exposure burden
cDE	cDE	cDEe	CcDE	Compatible alternative options

Representative rankings are shown for selected recipient Rh antigen-expression profiles. Rank 1 indicates the highest-priority donor antigen-profile option within the MCDA-derived ranking hierarchy. These rankings are intended to support prioritization among otherwise clinically acceptable donor antigen-profile options and should not be interpreted as estimates of individual alloimmunization risk. Detailed TOPSIS ranking results are provided in Supplementary Table S3. Antigen-expression profile labels indicate serologically detected antigen expression only and do not imply genotype or antigen homozygosity.

Examples of the Rh antigen matching score, storage-duration score, and integrated priority score are presented in Supplementary Table S4.

(4) Integrated Score and Inventory Adjustment

In routine practice, donor selection depends on both antigen matching and unit availability. A storage-duration score was therefore included as an auxiliary inventory adjustment factor. It was used to distinguish among clinically acceptable donor options with similar antigen matching.

The storage-duration score reflected inventory considerations only. It was not a measure of red blood cell storage lesion and was not used to predict post-transfusion immune-related outcomes (19–22). The complete workflow, from recipient-phenotype assessment and AHP weighting to TOPSIS-based donor ranking and inventory-assisted prioritization, is summarized in Figure 1.

**Figure 1:**
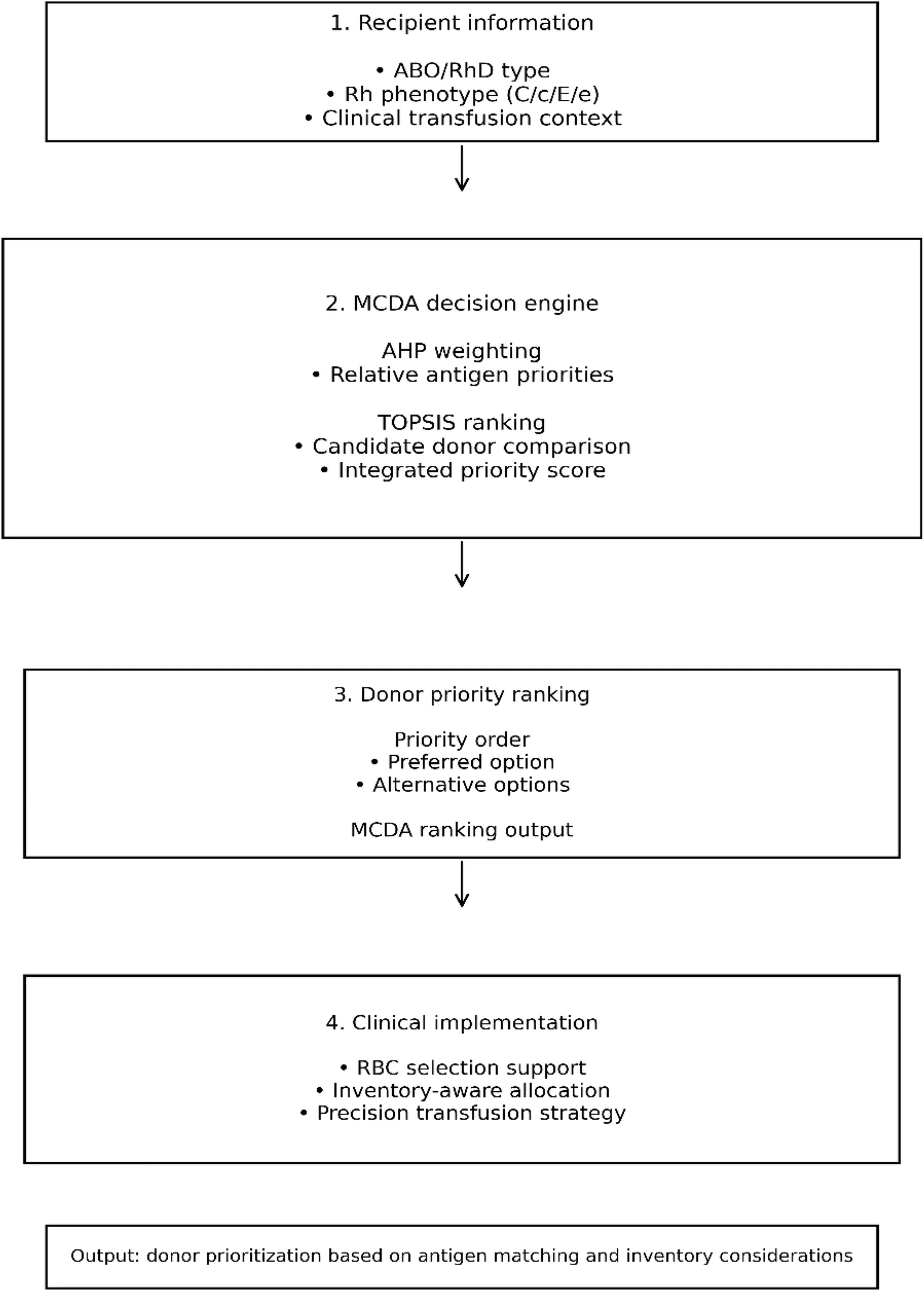
Conceptual framework of the MCDA-based Rh antigen-matching donor prioritization model. MCDA provides the overall structure for comparing multiple eligible donor options. AHP converts expert pairwise comparisons of the RhC, c, E, and e antigen-matching criteria into normalized criterion weights. TOPSIS then applies these weights to compare each donor option with theoretical positive and negative ideal solutions and calculates a closeness coefficient. A higher closeness coefficient indicates a higher donor priority. In the temporal exploratory evaluation, concordance between the actual transfused units and the MCDA-derived ranking was used to classify patients into high-match and low-match groups. AHP, analytic hierarchy process; MCDA, multi-criteria decision analysis; TOPSIS, technique for order preference by similarity to ideal solution.

### 2.3 Inventory-based auxiliary ranking module

In routine transfusion practice, donor-unit selection depends on both Rh antigen matching and inventory availability. To reflect this constraint, we added a storage-duration score to the MCDA framework as an optional inventory adjustment module.

The storage-duration score was used only to distinguish among otherwise acceptable donor units when Rh antigen matching was similar. It was not intended to measure the biological effects of red blood cell storage or to predict transfusion-related outcomes.

Integratedpriorityscore=Rhantigenmatchingscore×0.80+storage−durationscore×0.20.Both components were converted to normalized scores before integration. The integrated priority score was calculated using the normalized Rh antigen matching component and normalized storage-duration component as follows:

$Ii=0.80\times Mi+0.20\times SiI_{-}i=0.80\times M_{-}i+0.20\times S_{-}i$

where MiM_i represents the normalized Rh antigen matching component derived from the MCDA/TOPSIS-based ranking, and SiS_i represents the normalized storage-duration component used as an auxiliary inventory adjustment factor.

All integrated scores were calculated at the individual-patient level before group-level means and standard deviations were summarized.

For each recipient phenotype, TOPSIS was used to establish the relative priority order of eligible donor antigen-profile options. This recipient-specific priority hierarchy was subsequently represented by a prespecified Rh antigen matching score on a 0–10 scale. After normalization, the Rh antigen matching score was combined with the normalized storage-duration score to calculate the integrated priority score. The final donor-unit ranking was determined by the integrated priority score in descending order. The high-match/low-match classification was subsequently based on the concordance between the actual transfused unit and this final integrated-priority ranking, together with the prespecified criteria presented in Supplementary Table S5.

Rh antigen matching was the primary decision component and contributed 80% of the integrated score. The remaining 20% was assigned to the storage-duration component as an inventory-related adjustment. This ratio reflected the preferences of transfusion medicine specialists at our institution, who prioritized immunological matching while accounting for routine inventory management. The weights therefore.describe relative importance within this decision context and should not be interpreted as estimates of each component's effect on alloimmunization risk.

The storage-duration scoring rules are provided in Supplementary Table S6, and the weight-sensitivity analyses are presented in Supplementary Table S7.

### 2.4 Statistical analysis

Continuous variables were assessed for normality using the Shapiro–Wilk test. Normally distributed variables are presented as mea*n* ± standard deviation (SD) and were compared using the independent-samples t test. Non-normally distributed variables are presented as median and interquartile range (IQR) and were compared using the Mann–Whitney U test. Categorical variables are reported as number and percentage and were compared using the chi-square test or Fisher exact test, as appropriate.

Rh phenotype distributions were summarized descriptively as numbers and percentages. Hardy–Weinberg equilibrium was not assessed because the study population consisted of transfusion recipients rather than a population-based genetic cohort.

Rh phenotype distributions were summarized descriptively as numbers and percentages. Hardy–Weinberg equilibrium was not assessed because the study population consisted of transfusion recipients rather than a population-based genetic cohort.

For patients receiving multiple RBC units during the index transfusion episode, the integrated priority score and final donor-unit rank were determined separately for each transfused unit relative to the eligible donor inventory available at the time of issue. At the unit level, a transfused unit was classified as high-match if it ranked first, or if it ranked second with an Rh antigen matching score of at least 9 and an integrated priority score within 10% of the highest-ranked option. Units not meeting these criteria were classified as low-match. At the patient level, a worst-unit rule was applied: patients were classified as high-match only when all transfused units met the unit-level high-match criteria; patients receiving one or more low-match units were classified as low-match. For example, a patient receiving three units ranked first, first, and second would be classified as high-match if the second-ranked unit met both the Rh antigen matching score and integrated priority score criteria. In contrast, a patient receiving units ranked first and third would be classified as low-match because at least one unit did not meet the unit-level high-match criteria. These categories described concordance with the ranking framework and were not intended to define individual alloimmunization risk or transfusion suitability.

The prespecified primary outcome was newly detected irregular antibody positivity within 6 months after transfusion. Early irregular antibody positivity at 14–28 days, transfusion reactions, and subsequent crossmatch incompatibility were considered secondary outcomes. Continuous-score, Bayesian, and sensitivity analyses were considered exploratory or supportive. Because the number of new antibody events was limited, Firth-corrected logistic regression was used to reduce small-sample bias in maximum likelihood estimation (23, 24). Associations are reported as odds ratios (ORs) with 95% confidence intervals (CIs).

Covariates were prespecified on the basis of clinical relevance and potential confounding. The primary adjusted model included age, sex, previous transfusion history, and number of RBC units transfused. Recipient Rh phenotype was added in a sensitivity analysis to assess whether the main findings were influenced by the recipient antigen profile. Individual immune-responder status was not directly measured or incorporated into either the MCDA ranking framework or the outcome regression models because no standardized prospective measure of immune responsiveness was available in the retrospective clinical dataset. Previous transfusion history and the number of RBC units transfused were included as covariates to partially account for differences in prior and cumulative antigen exposure, but these variables should not be regarded as direct measures of individual immune responsiveness.

To limit information loss from categorical thresholds, the integrated MCDA priority score, Rh antigen matching score, and Rh antigen exposure space index (RAESI) were also analyzed as continuous variables. Their associations with newly detected irregular antibodies within 6 months were examined using logistic regression.

Stabilized inverse probability of treatment weighting (IPTW) was used as a sensitivity analysis to address residual confounding. The propensity score model included clinically relevant factors that could influence both donor-unit selection and irregular antibody formation. Covariate balance after weighting was assessed using standardized mean differences (SMDs), and the main association was re-estimated in the weighted cohort.

A Bayesian beta-binomial model was used to describe uncertainty in immune-related event rates between the high-match and low-match groups. This analysis was group based and exploratory; it was not intended for individual risk prediction or causal inference.

Weight-perturbation analyses were performed to assess the stability of donor rankings. Candidate donor antigen-profile options were reranked under alternative weighting scenarios,and consistency was evaluated using Spearman rank correlation and top-ranked recommendation agreement.

All analyses were performed in R version 4.3.2 (R Foundation for Statistical Computing, Vienna, Austria). All tests were two-sided, and *P* < 0.05 was considered statistically significant.

### 2.5 Bayesian group-level analysis of immune-related outcomes

A Bayesian beta-binomial model was used to further examine group-level differences in immune-related outcomes according to concordance with the MCDA donor ranking. By combining prior distributions with the observed data, the model provided posterior estimates of event-rate uncertainty under limited-event conditions (25).

Patients in the temporal exploratory cohort were classified as high match or low match according to the concordance between actual donor selection and the MCDA-derived priority ranking. The primary outcome was newly detected irregular antibody positivity within 6 months after transfusion.

For group g, let n_g denote the sample size, y_g the number of events, and *θ*_g the event probability. The sampling model was specified as:

y_g∼Binomial(n_g,θ_g)

θ_g∼Beta(1,1)

θ_g|y_g,n_g∼Beta(y_g+1,n_g−y_g+1)

The following quantities were estimated:

1. the posterior event rate in each group;
2. the posterior risk difference between the low-match and high-match groups, *Δθ* = *θ*_low−*θ*_high; and
3. the posterior probability that the event rate was higher in the low-match group, P(*θ*_low > *θ*_high | Data).

The posterior probability quantified the degree of support in the observed data for a higher event rate in the low-match group.

The Bayesian analysis was applied to the following outcomes: (1) newly detected irregular antibody positivity within 6 months; (2) early irregular antibody positivity 14–28 days after transfusion; (3) transfusion reactions; and (4). crossmatch incompatibility during follow-up.

## 3 Results

### 3.1 Cohort characteristics and distribution of Rh antigen phenotypes

A total of 313 patients who received RhD-positive red blood cell (RBC) transfusions were included in this study. The cohort consisted of 188 males (60.1%) and 125 females (39.9%), with a mean age of 57.6 ± 15.9 years. According to the concordance between the actual transfused donor RBC and the MCDA-derived donor priority ranking, patients were classified into the high-match group (*n* = 156) and the low-match group (*n* = 157).

The distributions of ABO blood groups, recipient Rh phenotypes, and major disease categories in the overall temporal exploratory cohort are summarized in Table 3. Baseline characteristics of the high-match and low-match groups are presented in Table 5. No statistically significant differences were observed between the two groups in age, sex, disease category, previous transfusion history, or number of RBC units transfused (all *P* > 0.05).

**Table 3:** Baseline characteristics and Rh antigen-expression profile distribution of the study cohort.

Variables	Overall cohort (*n* = 313)
Demographic characteristics	
Age, years	57.6 ± 15.9
Male sex, *n* (%)	188 (60.1)
Female sex, *n* (%)	125 (39.9)
ABO blood group, *n* (%)	
A	104 (33.2)
B	86 (27.5)
O	92 (29.4)
AB	31 (9.9)
Transfusion-related characteristics	
Previous transfusion history, *n* (%)	91 (29.1)
Number of previous transfusions, median (IQR)	1 (0–2)
Number of transfused RBC units, median (IQR)	3 (2–5)
Emergency transfusion, *n* (%)	73 (23.3)
Elective transfusion, *n* (%)	240 (76.7)
Disease category, *n* (%)	
Malignant tumor	103 (32.9)
Hematological disease	39 (12.5)
Cardiovascular disease	53 (16.9)
Gastrointestinal disease/surgery	47 (15.0)
Trauma/orthopedic disease	26 (8.3)
Other diseases	45 (14.4)
Rh status and phenotype combinations, *n* (%)	
RhD positive	313 (100.0)
Common Rh phenotype combinations, *n* (%)	
C + c + E−e+	145 (46.3)
C−c + E−e+	78 (24.9)
C + c + E + e+	56 (17.9)
C−c + E + e+	20 (6.4)
Other phenotypes	14 (4.5)

Continuous variables are presented as mean ± standard deviation or median (interquartile range), as appropriate, and categorical variables are presented as *n* (%). Rh antigen profiles were determined by serologic detection of C, c, E, and e antigens. The antigen-expression profile labels indicate serologically detected antigen expression only and do not imply genotype or antigen homozygosity. IQR, interquartile range; RBC, red blood cell.

The distribution of Rh antigen phenotypes in the study cohort is shown in Table 1. Among RhD-positive patients, the expression frequencies of RhC, c, E, and e antigens varied across different phenotypes, reflecting the Rh antigen characteristics of the transfusion population. These Rh phenotype data were used for subsequent development of the MCDA-based donor prioritization framework and sensitivity analyses.

Within the MCDA framework, patients were grouped according to the concordance between the actual transfused donor RBC and the model-derived donor priority ranking. The high-match and low-match classifications were used to evaluate the agreement between real-world transfusion practice and MCDA-based prioritization. These categories were not intended to define individual alloimmunization risk levels or clinical transfusion suitability.

### 3.2 AHP-derived weights for Rh antigen decision criteria

The relative weights of Rh antigen matching criteria within the MCDA framework were determined using the analytic hierarchy process (AHP), incorporating RhC, c, E, and e antigen distribution characteristics, previous immunological evidence, and clinical transfusion considerations.

The AHP pairwise comparison matrix was established by a panel of seven experts, including five specialists in transfusion medicine and two specialists in clinical laboratory medicine. The experts independently compared the relative importance of RhC, c, E, and e antigen matching criteria using the Saaty 1–9 scale. Criterion weights were subsequently calculated from the aggregated judgment matrix.

The final aggregated AHP pairwise comparison matrix, derived criterion weights, and consistency assessment are presented in Supplementary Tables S2A–C.

The maximum eigenvalue (*λ*max) of the judgment matrix was 4.0076. The consistency index (CI) was 0.0025, the random consistency index (RI) was 0.90, and the consistency ratio (CR) was 0.0028. The CR was below the prespecified threshold of 0.10, indicating acceptable consistency among expert judgments.

The final AHP-derived weights for Rh antigen criteria are presented in Table 4.

**Table 4:** MCDA-derived weight coefficients of Rh antigens (c, C, E, e) in the integrated donor prioritization scoring model.

**Rh antigen**	**Weight**
c	0.5528
E	0.2687
e	0.1202
C	0.0583

Among the evaluated criteria, the c antigen received the highest weight, followed by E, e, and C antigens. These weights represented the relative contribution of each criterion within the donor prioritization framework and were not intended to re-estimate the absolute biological immunogenicity of individual Rh antigens.

Using these AHP-derived weights, TOPSIS was subsequently applied to rank donor antigen-profile options for different recipient Rh phenotypes, as described in Section 3.3.

### 3.3 MCDA-based prioritization of Rh antigen-matched donor units

The MCDA framework integrated RhC, c, E, and e antigen matching characteristics with AHP-derived weights, and TOPSIS was subsequently applied to rank donor antigen-profile options for different recipient Rh phenotypes.

For each recipient phenotype, candidate donor units were assigned a distinct priority order. When a fully matched Rh antigen profile was available, the corresponding donor antigen-profile option consistently ranked first. In the absence of a fully matched option, donor antigen-profile options associated with fewer additional Rh antigen exposures generally received higher rankings, whereas donor antigen-profile options involving greater potential exposure to recipient-missing antigens ranked lower.

Representative donor antigen-profile ranking results are shown in Table 2. For recipients with the CDe antigen-expression profile, the CDe donor antigen-profile option ranked first, followed by the CcDe and CcDEe options. For recipients with the CcDEe antigen-expression profile, the CcDEe donor antigen-profile option received the highest priority, followed by the CDEe and cDEe options. These rankings followed the predefined MCDA principle of prioritizing reduced potential Rh antigen exposure.

Detailed TOPSIS priority scores and Rh antigen exposure space index (RAESI) values for the donor antigen-profile options are provided in Supplementary Table S3.

An Rh antigen matching score was subsequently derived from the MCDA ranking system. Fully matched donor antigen-profile options received the highest scores, whereas partially matched donor antigen-profile options were scored according to the extent of additional antigen exposure. Lower scores were assigned to schemes with greater exposure to recipient-missing antigens. The scoring system was used to compare relative priorities among clinically acceptable donor options and was not intended to estimate individual alloimmunization risk.

A storage-duration component was incorporated as an auxiliary inventory-related factor in the integrated priority score. Sensitivity analysis showed that removing this component produced rankings highly consistent with those from the full MCDA framework. Rh antigen matching remained the dominant determinant of donor prioritization, whereas storage duration mainly contributed secondary adjustment under inventory constraints.

The TOPSIS results represented the antigen-based priority hierarchy. After incorporation of the auxiliary storage-duration component, candidate donor units were finally ranked according to their integrated priority scores.

### 3.4 Weight sensitivity analysis

Sensitivity analyses were performed to evaluate the impact of AHP weight selection on MCDA ranking results. In addition to the reference AHP weighting scheme, alternative weighting strategies were assessed, including a conservative weighting approach and an increased weighting for the c-antigen criterion.

TOPSIS rankings were recalculated under each weighting scenario. Representative results for different Rh phenotypes are shown in Supplementary Table S7. Across the representative Rh phenotypes shown in Supplementary Table S7, the top-ranked donor antigen-profile option remained unchanged under the evaluated weighting strategies.

Ranking stability was further evaluated using Spearman rank correlation coefficients. Across the representative Rh phenotypes, the alternative weighting strategies showed strong agreement with the reference AHP model, with Spearman *ρ* values ranging from 0.92 to 0.96.

Although the AHP weights were derived from expert judgment, the donor rankings remained stable under the evaluated weight perturbation scenarios. These findings support the robustness of the MCDA prioritization framework to reasonable variations in criterion weighting.

### 3.5 Temporal exploratory evaluation

#### 3.5.1 Baseline characteristics of the temporal exploratory cohort

Baseline characteristics of the high-match group (*n* = 156) and low-match group (*n* = 157) are presented in Table 5.

**Table 5:** Baseline characteristics of patients in the high-match and low-match groups in the temporal exploratory cohort.

Variables	High-match group (*n* = 156)	Low-match group (*n* = 157)	*P* value
Age, years	56.8 ± 15.7	58.3 ± 16.2	0.43
Male sex, *n* (%)	92 (59.0)	96 (61.1)	0.71
Disease category, *n* (%)			
Malignant tumor	48 (30.8)	55 (35.0)	0.44
Hematological disease	18 (11.5)	21 (13.4)	0.63
Cardiovascular disease	28 (17.9)	25 (15.9)	0.65
Gastrointestinal disease/surgery	25 (16.0)	22 (14.0)	0.64
Trauma/orthopedic disease	14 (9.0)	12 (7.6)	0.68
Other diseases	23 (14.7)	22 (14.0)	0.87
Previous transfusion history, *n* (%)	42 (26.9)	49 (31.2)	0.42
Number of previous transfusions, median (IQR)	0 (0–2)	1 (0–2)	0.31
Number of transfused RBC units, median (IQR)	2 (2–4)	3 (2–5)	0.08
Emergency transfusion, *n* (%)	31 (19.9)	42 (26.8)	0.16
Elective transfusion, *n* (%)	125 (80.1)	115 (73.2)	0.16
Hospital department, *n* (%)			
Surgery department	62 (39.7)	58 (36.9)	0.63
Internal medicine department	53 (34.0)	61 (38.9)	0.39
Hematology department	18 (11.5)	21 (13.4)	0.63
Emergency/ICU	23 (14.7)	17 (10.8)	0.32

Continuous variables are presented as mean ± standard deviation or median (interquartile range), and categorical variables are presented as *n* (%). Comparisons were performed using Student's t test, Mann–Whitney U test, *χ*^2^ test, or Fisher's exact test, as appropriate. IQR, interquartile range; ICU, intensive care unit; RBC, red blood cell.

No statistically significant differences were observed between the groups in age, sex, disease category, malignancy or hematologic disease status, previous transfusion history, number of RBC units transfused, proportion of emergency transfusions, or hospital department distribution (all *P* > 0.05).

#### 3.5.2 Primary and secondary outcome analyses

Patients were classified into high-match (*n* = 156) and low-match (*n* = 157) groups according to the concordance between the actual transfused unit and the final donor-unit ranking determined by the integrated priority score. Comparisons of donor-unit characteristics and MCDA priority scores are shown in Table 6, whereas immune-related outcomes are presented in Table 7.

**Table 6:** Comparison of transfused-unit characteristics and MCDA priority scores between groups.

Variable	High-match group (*n* = 156)	Low-match group (*n* = 157)	Statistic	*P* value
Transfused-unit characteristics, *n* (%)				
Phenotype-identical transfusion	86 (55.1)	0 (0.0)	—	<0.001
Compatible transfusion	70 (44.9)	62 (39.5)	—	0.335
Lower-priority transfusion	0 (0.0)	95 (60.5)	—	<0.001
MCDA priority scores				
Rh antigen matching score, raw 0–10 scale	9.12 ± 0.78	8.36 ± 1.06	t = 7.24	<0.001
Normalized Rh antigen matching component, 0–1 scale	0.912 ± 0.078	0.836 ± 0.106	t = 7.24	<0.001
Normalized storage-duration component, 0–1 scale	0.76 ± 0.19	0.61 ± 0.23	t = 6.29	<0.001
Integrated priority score, 0–1 scale	0.88 ± 0.08	0.79 ± 0.10	t = 8.79	<0.001

Data are presented as *n* (%) or mean ± SD, as appropriate.

**Table 7:** Association between patient-level MCDA matching status and immune-related outcomes.

Panel A. Firth-corrected logistic regression analysis for newly detected irregular antibody positivity within 6 months			
Variable	OR	95% CI	*P* value
High-match group	**0**.**29**	**0.10**–**0.82**	**0**.**020**
Age, per year	1.01	0.98–1.03	0.412
Male sex	0.94	0.41–2.16	0.881
Previous transfusion history	1.06	0.92–1.22	0.418
Number of transfused RBC units	1.08	0.98–1.19	0.126

Panel B. Bayesian beta-binomial analysis of immune-related outcomes according to MCDA matching status			
Outcome	High-match group (*n* = 156)	Low-match group (*n* = 157)	Posterior probability (Low-match > High-match)
Newly detected irregular antibody positivity within 6 months	5 (3.2%)	18 (11.5%)	99.7%
Early irregular antibody positivity at 14–28 days	3 (1.9%)	13 (8.3%)	99.5%
Transfusion-related reactions	2 (1.3%)	9 (5.7%)	98.2%
Subsequent crossmatch incompatibility during follow-up	1 (0.6%)	10 (6.4%)	99.7%

Panel A presents Firth-corrected logistic regression evaluating the association between MCDA-based high-match status and newly detected irregular antibody positivity within 6 months. Odds ratios represent association estimates and should not be interpreted as individual risk-prediction parameters. Panel B presents Bayesian beta-binomial analyses using a noninformative Beta (1,1) prior. Posterior probability represents the probability that the event rate in the low-match group was higher than that in the high-match group under the specified model assumptions. These analyses were exploratory and were not intended for individual risk prediction or causal inference.

Bold values indicate statistically significant results (*P* < 0.05).

Compared with the low-match group, the high-match group had a higher proportion of phenotype-identical transfusions (55.1% vs. 0%; *P* < 0.001) and a lower proportion of lower-priority transfusions (0% vs. 60.5%; *P* < 0.001). The proportion of compatible transfusions did not differ significantly between groups (44.9% vs. 39.5%; *P* = 0.335).

The high-match group had a higher raw Rh antigen matching score than the low-match group (9.12 ± 0.78 vs. 8.36 ± 1.06; *P* < 0.001), corresponding to normalized Rh antigen matching scores of 0.912 ± 0.078 and 0.836 ± 0.106, respectively (*P* < 0.001). The normalized storage-duration score was also higher in the high-match group (0.76 ± 0.19 vs. 0.61 ± 0.23; *P* < 0.001). Consequently, the integrated priority score was higher in the high-match group than in the low-match group (0.88 ± 0.08 vs. 0.79 ± 0.10; *P* < 0.001) (Table 5).

For the prespecified primary outcome, newly detected irregular antibodies within 6 months after transfusion occurred in 5 patients (3.2%) in the high-match group and 18 patients (11.5%) in the low-match group (*P* = 0.008). The specificities of the newly detected irregular antibodies in the two groups are presented in Supplementary Table S8. Early irregular antibody positivity at 14–28 days (1.9% vs. 8.3%; *P* = 0.018) and subsequent crossmatch incompatibility during follow-up (0.6% vs. 6.4%; *P* = 0.010) were also less frequent in the high-match group. The difference in transfusion reaction rates did not reach statistical significance (1.3% vs. 5.7%; *P* = 0.061).

Firth-corrected logistic regression showed that high-match status was associated with lower odds of newly detected irregular antibodies (OR, 0.29; 95% CI, 0.10–0.82; *P* = 0.020). The direction of the association remained consistent after stabilized inverse probability of treatment weighting and propensity score matching. Residual confounding, however, could not be excluded.

Under a noninformative Beta(1,1) prior, Bayesian beta-binomial analysis yielded a 99.7% posterior probability that the 6-month irregular antibody positivity rate was higher in the low-match group than in the high-match group. For the secondary outcomes, the posterior probabilities that the event rates were higher in the low-match group were 99.5% for early irregular antibody positivity, 98.2% for transfusion reactions, and 99.7% for crossmatch incompatibility within 6 months.

In this temporal exploratory cohort, concordance between actual donor selection and MCDA-based prioritization was associated with immune-related outcomes. These findings do not establish that the MCDA framework reduces alloimmunization risk at the individual-patient level. Larger multicenter prospective studies are needed to confirm the observed associations.

The Rh antigen matching score was derived from the MCDA/TOPSIS-based ranking system and initially represented on a 0–10 clinical interpretation scale. The normalized Rh antigen matching component was calculated as:

Mi=Mraw,i/10M_i=M_{raw,i}/10

The storage-duration component was independently normalized according to the predefined inventory-adjustment scoring rule and expressed as a 0–1 scale value (SiS_i).

The integrated priority score was calculated at the individual donor-unit level before patient-level classification and group-level summarization:

$$Ii=0.80\times Mi+0.20\times SiI_{-}i=0.80\setminus times\;M_{i}+0.20\setminus times\;S_{-}i$$

where MiM_i represents the normalized Rh antigen matching component and SiS_i represents the normalized storage-duration component.

For patients receiving multiple RBC units, high-match status required all transfused units to meet the prespecified unit-level high-match criteria; receipt of at least one unit not meeting these criteria resulted in low-match classification.

### 3.6 Association between continuous MCDA scores and newly detected irregular antibodies

To assess the continuous relationship between MCDA ranking metrics and post-transfusion immune-related outcomes, and to reduce information loss associated with categorical thresholds, the MCDA integrated priority score, Rh antigen matching score, and Rh antigen exposure space index (RAESI) were entered into logistic regression models as continuous variables. Their associations with newly detected irregular antibodies within 6 months were then examined.

In univariable logistic regression, a higher MCDA integrated priority score was associated with lower odds of newly detected irregular antibodies within 6 months (OR, 0.86; 95% CI, 0.76–0.97; *P* = 0.014). A similar inverse association was observed for the Rh antigen matching score (OR, 0.78; 95% CI, 0.64–0.95; *P* = 0.012). By contrast, a higher RAESI was associated with greater odds of newly detected irregular antibodies (OR, 1.32; 95% CI, 1.08–1.62; *P* = 0.006).

After adjustment for age, sex, previous transfusion history, and number of RBC units transfused, the MCDA integrated priority score remained inversely associated with newly detected irregular antibodies (adjusted OR, 0.88; 95% CI, 0.78–0.99; *P* = 0.031). The Rh antigen matching score also retained an inverse association (adjusted OR, 0.81; 95% CI, 0.66–0.98; *P* = 0.028), whereas RAESI remained positively associated with the outcome (adjusted OR, 1.25; 95% CI, 1.03–1.54; *P* = 0.026).

The direction of these continuous-score associations was consistent with the high-match versus low-match analysis. This suggests that the observed findings were not driven solely by the selected classification thresholds, but also reflected a graded relationship between MCDA ranking metrics and Rh antigen matching.

### 3.7 Exploratory association between MCDA matching status and immune-related outcomes

Recipients were classified into high-match and low-match groups according to the MCDA-derived priority ranking. For patients receiving multiple RBC units during the index transfusion episode, each transfused unit was evaluated separately. At the unit level, a unit was classified as high-match if it ranked first, or if it ranked second with an Rh antigen matching score of at least 9 and an integrated priority score difference of no more than 10% from the highest-ranked option. Units ranked third or lower, units with an Rh antigen matching score of 7 or lower, and Rank 2 units that did not meet the near-optimal criteria were classified as low-match. At the patient level, a worst-unit rule was applied: patients were classified as high-match only when all transfused units met the unit-level high-match criteria; receipt of at least one low-match unit resulted in classification as low-match.

These categories were used to compare immune-related outcomes across different levels of concordance with MCDA-based prioritization. They were not intended to define clinical transfusion risk.

During follow-up, newly detected irregular antibodies occurred in 3.2% (5/156) of patients in the high-match group and 11.5% (18/157) of those in the low-match group (*P* = 0.008). After adjustment for age, sex, previous transfusion history, and number of RBC units transfused, Firth-corrected logistic regression showed that high-match status was associated with lower odds of newly detected irregular antibodies (OR, 0.29; 95% CI, 0.10–0.82; *P* = 0.020) (Table 6). Early irregular antibody positivity was also less frequent in the high-match group than in the low-match group (1.9% vs. 8.3%; *P* = 0.018), as was crossmatch incompatibility during follow-up (0.6% vs. 6.4%; *P* = 0.010). The difference in transfusion-related reactions did not reach statistical significance (1.3% vs. 5.7%; *P* = 0.061).

Sensitivity analyses were performed to assess the stability of the grouping criteria. The high-match group was redefined using a Rank 1-only strategy, alternative Rh antigen matching score thresholds, including a cutoff of at least 8, and different allowable score differences from the top-ranked option. Across these alternative definitions, the direction of the association between MCDA matching status and newly detected irregular antibodies remained broadly consistent (Supplementary Table S5).

Potential exposure misclassification among patients receiving multiple RBC units was additionally assessed using a majority-rule definition, under which patients were classified as high-match when at least 50% of the transfused units met the unit-level high-match criteria. Under this alternative definition, 18 patients were reclassified from the low-match group to the high-match group, resulting in 174 patients in the high-match group and 139 patients in the low-match group. Newly detected irregular antibodies occurred in 7 patients (4.0%) and 16 patients (11.5%), respectively. The association remained directionally consistent with the primary worst-unit analysis (Firth-corrected OR, 0.34; 95% CI, 0.13–0.82; *P* = 0.018) (Supplementary Table S5).

Bayesian beta-binomial analysis further showed that, under the prespecified model assumptions, the posterior probability of a higher rate of newly detected irregular antibodies in the low-match group was at least 98%.

In this temporal exploratory cohort, MCDA matching status was associated with post-transfusion immune-related outcomes. However, given the study design and limited number of events, these findings do not establish that MCDA-based matching reduces alloimmunization risk. Confirmation in larger, multicenter prospective studies is required.

### 3.8 Sensitivity and robustness analyses

To assess the robustness of the association between MCDA matching status and immune-related outcomes, multiple sensitivity analyses were performed.

When patients were classified solely according to the Rh antigen matching score, the high-match group continued to show a lower tendency for immune-related events. In contrast, classification based only on the storage-duration score did not reveal a statistically significant difference between groups. Rankings generated using different score integration approaches, including multiplicative and weighted-sum models, showed high concordance. These findings suggest that Rh antigen matching was the primary determinant of donor prioritization, whereas the storage-duration component mainly served as an auxiliary adjustment under inventory constraints.

Under the assumptions of a beta-binomial model with a noninformative Beta(1,1) prior, the Bayesian group-level analysis showed a posterior probability of 99.7% that the 6-month incidence of newly detected irregular antibodies was higher in the low-match group than in the high-match group. This probability reflected the uncertainty regarding the direction of group-level event-rate differences under the observed data and model assumptions.

The Bayesian posterior probability was used to describe differences in event rates between groups and should not be interpreted as evidence of definite clinical benefit associated with high-match transfusion selections. It also does not establish that the MCDA framework directly reduces alloimmunization risk or predicts individual immune responses.

The sensitivity analysis results were consistent with the findings from Firth-corrected logistic regression, stabilized IPTW, and propensity score matching analyses, supporting a stable observational association between MCDA matching status and immune-related outcomes. However, given the single-center, retrospective, and exploratory nature of this evaluation, these findings require further confirmation in larger multicenter prospective studies.

Bayesian analyses of additional immune-related outcomes are presented in Table 6 (Panel B).

## 4 Discussion

### 4.1 Main findings

This study developed an MCDA-based framework for prioritizing Rh antigen-matched RBC units and performed a temporal exploratory evaluation in a real-world cohort of patients receiving RhD-positive RBC transfusions. The main contribution of this work is that it extends conventional Rh antigen matching from a binary compatibility assessment to a structured ranking problem among multiple clinically acceptable donor options.

The framework integrated RhC, c, E, and e antigen matching characteristics, expert-derived AHP weights, TOPSIS ranking, and inventory-related considerations. By combining these factors within a quantitative decision structure, the framework enabled transparent comparison and prioritization of donor antigen-profile options**.** MCDA has been increasingly applied in healthcare decision-making because it provides a systematic approach for balancing multiple competing criteria (12, 13).

The framework generated consistent priority rankings across different recipient Rh phenotypes and remained stable under various weight perturbation scenarios. In the temporal exploratory cohort, patients whose actual transfused donor RBCs showed greater concordance with MCDA-derived prioritization had fewer newly detected irregular antibodies within 6 months. Similar associations were observed in Firth-corrected logistic regression, stabilized IPTW, propensity score matching, and Bayesian analyses. Newly detected irregular antibody positivity within 6 months was the prespecified primary outcome, whereas findings from the secondary outcomes, continuous-score models, Bayesian analyses, and sensitivity analyses should be regarded as supportive and hypothesis-generating.

These findings should be interpreted cautiously. The observed association reflects the relationship between actual donor selection and MCDA ranking concordance, rather than a causal effect of the MCDA framework itself. RBC alloimmunization is influenced by multiple factors, including antigen exposure, transfusion burden, immune status, inflammatory conditions, and genetic background (3, 4). Therefore, this study evaluated the feasibility, interpretability, and potential application of MCDA as a structured transfusion decision-support tool rather than its clinical effectiveness as an intervention.

Precision transfusion strategies are increasingly moving beyond ABO/RhD compatibility toward more structured use of extended antigen profiles. In the present framework, recipient specificity was limited to the recipient's RhC, c, E, and e phenotype and the corresponding potential exposure to recipient-missing antigens. Unlike conventional Rh antigen matching, which generally categorizes donor units as identical, compatible, or alternative, the MCDA framework generates a weighted priority ranking among multiple units that already meet routine transfusion requirements. Unlike inventory-based allocation, it retains Rh antigen matching as the dominant decision component and uses storage duration only as an auxiliary discriminator among otherwise similarly matched units. The framework should therefore be interpreted as a recipient-phenotype–specific prioritization tool rather than a comprehensive patient-specific risk model.

### 4.2 Clinical implications of the decision framework

The MCDA framework was developed to address a practical challenge in transfusion medicine: selecting among multiple RBC units that already meet routine compatibility requirements. When several ABO/RhD-compatible and crossmatch-compatible units are available, donor selection often relies on empirical principles such as identical matching or minimizing additional antigen exposure. However, current Rh matching strategies mainly focus on compatibility assessment and high-risk patient management, while systematic approaches for ranking multiple acceptable options remain limited (1–4).

In this study, AHP converted immunological evidence, regional antigen distribution characteristics, and expert clinical judgment into structured decision weights. TOPSIS was then used to rank donor antigen-profile options according to these criteria. The advantage of MCDA lies in its ability to integrate heterogeneous information while improving transparency and interpretability in complex decision processes (12–15).

The AHP-derived weights should be interpreted as the relative contribution of each criterion within the prioritization framework rather than direct estimates of antigen immunogenicity. Accordingly, the framework supports comparative decision-making among donor options but does not replace established immunohematological assessment methods (5, 6).

The temporal exploratory evaluation showed an association between higher MCDA ranking concordance and lower rates of newly detected irregular antibodies. Because donor allocation was not prospectively guided by MCDA, these findings represent exploratory evidence of potential stratification value rather than proof of clinical benefit. Previous studies have demonstrated that extended Rh antigen matching may reduce alloimmunization in selected populations, although implementation depends on patient characteristics, transfusion requirements, and resource availability (3–6).

The storage-duration component was included as an auxiliary adjustment factor to reflect inventory constraints. It was not intended to model RBC storage lesions or biological effects of storage. RBC storage-related changes involve complex metabolic and structural alterations beyond the scope of this framework (19–21). Sensitivity analyses showed that removing this component had little effect on donor rankings, confirming that Rh antigen matching remained the primary driver of prioritization.

The potential clinical value of this framework lies in supporting donor selection when multiple compatible RBC units are available. By converting multidimensional antigen information into a transparent and reproducible ranking, the framework may improve the consistency of donor-unit selection and facilitate explicit trade-offs when antigen matching is similar but inventory conditions differ. However, because Rh antigen matching was the dominant component of the model, the present study does not establish that the integrated MCDA score provides incremental predictive value beyond conventional antigen matching. Its proposed added value is primarily operational and decision-oriented, including standardized prioritization, transparent documentation, and secondary differentiation among otherwise similarly matched units. Formal comparisons of discrimination, reclassification, clinical net benefit, allocation efficiency, and inventory utilization will be required in future prospective studies.

Overall, this framework represents a methodological transition from binary compatibility assessment toward quantitative prioritization among acceptable donor options. Further multicenter prospective studies are required to determine its clinical utility.

### 4.3 Interpretation of the Bayesian group-level analysis

This study incorporated Bayesian group-level analysis as a complementary approach to evaluate uncertainty in immune-related event rates between groups with different levels of MCDA ranking concordance. Bayesian methods combine prior information with observed data to estimate posterior uncertainty and may be useful in exploratory analyses involving limited events (23).

In this study, Bayesian analysis was used only to characterize group-level differences. It was not intended to develop an individual alloimmunization risk model or establish causal relationships between donor selection and clinical outcomes. Therefore, posterior probabilities should be interpreted as statistical support for the observed direction of group differences under the specified assumptions, rather than as individual risk estimates.

The Bayesian findings were consistent with Firth-corrected logistic regression, stabilized IPTW, and propensity score matching analyses, supporting the reproducibility of the observed association between MCDA ranking concordance and immune-related outcomes. However, the limited number of events requires confirmation in larger and more diverse populations.

### 4.4 Interpretation of the association between MCDA matching concordance and immune outcomes

The study observed an association between high MCDA matching status and lower rates of newly detected irregular antibodies. However, this finding should not be interpreted as evidence that MCDA-guided selection reduces alloimmunization risk.

The high-match and low-match classifications were derived from the predefined MCDA ranking system. Therefore, the observed association may primarily reflect differences in Rh antigen matching and antigen exposure rather than an independent effect of applying the framework.

The purpose of this study was to explore the feasibility and potential value of an MCDA-based decision-support approach in routine transfusion practice, rather than to validate a predictive model. The findings should therefore be considered hypothesis-generating and require confirmation through prospective multicenter investigations.

### 4.5 Limitations and future perspectives

This study developed an MCDA-based framework for prioritizing Rh antigen-matched RBC donor units and performed a temporal exploratory evaluation to assess its applicability in a real-world transfusion setting. Several limitations should be acknowledged.

First, this study was not designed as a prospective evaluation of clinical outcomes after MCDA-guided transfusion allocation. Instead, it assessed the concordance between actual donor selection and MCDA-derived ranking under routine transfusion practice and explored its observational association with post-transfusion immune-related outcomes. Therefore, the findings should not be interpreted as evidence that the MCDA framework itself reduces alloimmunization risk. Rather, the framework may provide a structured and transparent approach for integrating existing Rh antigen matching principles and prioritizing multiple acceptable donor options.

Continuous-variable analyses further showed that the MCDA integrated priority score and Rh antigen matching score were associated with immune-related outcomes in a graded manner rather than solely through predefined categorical thresholds. However, because the MCDA scoring system was primarily constructed from Rh antigen matching characteristics, these findings should be interpreted as supporting the consistency of the ranking framework rather than demonstrating additional predictive value beyond antigen matching itself.

Second, the high-match and low-match classifications were derived from the predefined MCDA ranking system. Therefore, these categories reflected both the concordance between actual transfused donor RBCs and model-derived priorities and the underlying level of Rh antigen matching. Although weight perturbation analyses, alternative classification thresholds, and sensitivity analyses supported the robustness of the findings, and adjustment for recipient Rh phenotype showed consistent associations, the limited number of outcome events prevented complete exclusion of residual confounding. Potential sources include overlap between the defined exposure and antigen matching status, as well as unmeasured factors such as disease severity, transplantation history, and pregnancy history in female patients. Future studies should evaluate the incremental value of the framework beyond conventional Rh matching strategies using continuous MCDA scores, antigen exposure metrics, and formal incremental prediction analyses.

Third, this framework was developed within a single regional blood supply system, and ethnicity and genetic ancestry were not included in the analysis. Because Rh antigen distributions vary among populations, external application may require validation and recalibration using local recipient and donor data.

Fourth, the storage-duration score was incorporated as an auxiliary inventory-related factor to simulate the influence of blood availability on donor selection in routine practice. It was not intended to evaluate RBC storage lesions or predict transfusion-related clinical outcomes. The weighting scheme and scoring thresholds for this component were derived from institutional inventory management practices and expert judgment, and their generalizability requires validation in different blood supply systems. Future work may integrate dynamic inventory management models, supply–demand prediction algorithms, and patient-specific immune characteristics to further optimize donor selection strategies.

Fifth, the framework was limited to serologically determined RhC, c, E, and e antigens and did not account for variant Rh antigens or other clinically relevant blood group systems. Future studies should incorporate molecular genotyping and evaluate broader antigen profiles in ethnically diverse populations.

Individual immune-responder status was also not directly assessed. Although previous transfusion history and transfusion burden were included in the adjusted analyses, these variables cannot fully capture biological differences in immune responsiveness. Residual confounding arising from unmeasured inflammatory, genetic, immunological, and treatment-related factors therefore remains possible. Future prospective studies should incorporate more detailed immune-response phenotyping and longitudinal antibody history.

Future multicenter prospective implementation studies are needed to evaluate the feasibility, clinical acceptance, and long-term value of deploying MCDA-based donor prioritization in routine transfusion workflows. Integration with Rh genotyping, large-scale data analytics, and artificial intelligence approaches may further enhance personalized and intelligent RBC transfusion decision-making.

## Data Availability

The original contributions presented in the study are included in the article/Supplementary Material, further inquiries can be directed to the corresponding authors.
