# Supplementary material for: Development and temporal exploratory evaluation of a multi-criteria decision analysis framework for Rh antigen-matched red blood cell donor prioritization
Source: Front Med (Lausanne). 2026 Oct 16;13:1962032. doi: 10.3389/fmed.2026.1962032 (PMC13719416; doi:10.3389/fmed.2026.1962032)
Supplement: Supplementary file 1 [file Table1.docx]

**Supplementary Table S1. Immunogenicity and relative immunogenicity of Rh antigens**

|  | **D** | **C** | **c** | **E** | **e** |
| --- | --- | --- | --- | --- | --- |
| Immunogenicity (%) | 50.00 | 0.11 | 2.05 | 1.69 | 0.56 |
| Relative immunogenicity (%) | 2.66 | 0.02 | 0.46 | 0.33 | 0.11 |

**Footnote**
Relative immunogenicity values were calculated from previously reported antigen-specific immunogenicity data and were used only as supportive evidence during expert judgment. These values were not directly incorporated as MCDA weights, which were derived from the AHP pairwise comparison matrix. RhD was included for contextual comparison because the study focused on RhD-positive red blood cell transfusion recipients; however, RhD was not included as a decision criterion in the MCDA framework.

**Supplementary Table S2. Descriptive analysis of Rh antigen phenotype/genotype distribution in the study cohort**

| **Rh phenotype/genotype** | **Observed n (%)** | **Expected n (%)** | **χ² contribution** | **P value** |
| --- | --- | --- | --- | --- |
| CCDE− | 19 (0.55) | 19.0000 (0.55) | 0.0000 | 0.0042 |
| CCDee | 1480 (42.84) | 1480.8997 (42.86) | 0.0005 | 0.6493 |
| CcDE− | 1255 (36.32) | 1211.6881 (35.07) | 1.5482 | 0.2656 |
| CcDee | 339 (9.81) | 339.4331 (9.82) | 0.0006 | 0.0244 |
| ccDE− | 339 (9.81) | 382.3119 (11.07) | 4.9068 | 0.0510 |
| ccDee | 12 (0.35) | 10.6671 (0.31) | 0.1665 | 0.0054 |
| ccdee | 9 (0.26) | 9.0000 (0.26) | 0.0000 | 0.0000 |
| CcdE− | 0 (0.00) | 0.0000 (0.00) | – | 0.0000 |
| CCdee | 1 (0.03) | 0.0000 (0.00) | – | 0.6650 |
| ccdE− | 0 (0.00) | 0.0000 (0.00) | – | 0.3350 |
| Ccdee | 1 (0.03) | 1.8997 (0.05) | 0.4261 | 0.9436 |
| CCdE− | 0 (0.00) | 0.0000 (0.00) | – | 0.0564 |
| **Total** | **3455 (100.0)** | **3455 (100.0)** | **7.0487** | **0.2698** |

**Footnote**
Observed Rh phenotype/genotype frequencies are presented with corresponding expected frequencies for descriptive comparison. Because this cohort consisted of transfusion recipients rather than a population-based genetic sample, these analyses were not intended for evaluation of Hardy–Weinberg equilibrium or population genetic inference.

**Supplementary Table S3. TOPSIS priority score and Rh antigen exposure space index (RAESI) of donor Rh antigen combination schemes**

| **Donor scheme** | **C** | **c** | **E** | **e** | **TOPSIS priority score** | **RAESI** |
| --- | --- | --- | --- | --- | --- | --- |
| A1 | 1 | 0 | 0 | 1 | 0.82 | 0.12 |
| A2 | 1 | 0 | 1 | 1 | 0.79 | 0.18 |
| A3 | 1 | 1 | 0 | 1 | 0.75 | 0.22 |
| A4 | 0 | 1 | 0 | 1 | 0.63 | 0.35 |
| A5 | 0 | 1 | 1 | 1 | 0.58 | 0.41 |
| A6 | 1 | 1 | 1 | 1 | 0.71 | 0.28 |
| A7 | 0 | 0 | 1 | 1 | 0.46 | 0.63 |
| A8 | 0 | 0 | 0 | 1 | 0.31 | 0.78 |
| A9 | 0 | 0 | 1 | 0 | 0.27 | 0.81 |

**Footnote:**

C, c, E, and e represent Rh antigen status of donor red blood cells.

Binary coding was applied to represent antigen exposure characteristics within the MCDA decision matrix. A value of 1 indicates the presence of the corresponding Rh antigen, whereas 0 indicates absence.

The TOPSIS priority score represents the relative preference value of each donor antigen combination within the MCDA framework. Higher values indicate a higher priority ranking among candidate donor schemes.

The Rh antigen exposure space index (RAESI) describes the relative position of each donor antigen combination within the multidimensional antigen exposure decision space. Higher RAESI values indicate greater antigen exposure burden relative to the recipient-compatible reference state.

RAESI is a relative decision-space indicator and should not be interpreted as an estimate of absolute alloimmunization probability or an individual patient risk prediction metric.

**Supplementary Table S4.MCDA-based Rh antigen matching score and inventory-adjusted priority score of donor schemes**

| **Donor scheme** | **Rh antigen matching score** | **Storage duration score** | **Integrated priority score** |
| --- | --- | --- | --- |
| A1 | 10.0 | 10.0 | 10.0 |
| A2 | 9.0 | 10.0 | 9.2 |
| A3 | 9.0 | 10.0 | 9.2 |
| A4 | 8.0 | 10.0 | 8.4 |
| A5 | 8.0 | 10.0 | 8.4 |
| A6 | 7.0 | 10.0 | 7.6 |
| A7 | 6.0 | 10.0 | 6.8 |
| A8 | 4.0 | 10.0 | 5.2 |
| A9 | 2.0 | 10.0 | 3.6 |

**Footnote:**
The integrated priority score was calculated as: Rh antigen matching score × 0.80 + storage duration score × 0.20. Rh antigen matching score was considered the primary decision dimension, whereas storage duration score was incorporated as an auxiliary inventory optimization factor. The integrated priority score represents a relative ranking indicator within the MCDA framework and should not be interpreted as a biological predictor of transfusion outcome or red blood cell storage lesion.

**Supplementary Table S5. Operational storage-duration adjustment rules for inventory-assisted donor prioritization**

| **Blood group type** | **Storage duration (days)** | **Storage score** |
| --- | --- | --- |
| A/B/O | N ≤ 5 | 10.0 |
| A/B/O | N > 5 | 12 − 0.4N |
| AB | N ≤ 10 | 10.0 |
| AB | N > 10 | 15 − 0.5N |

**Footnote**

N represents the storage duration of donor red blood cell units (days). The storage duration score was incorporated into the MCDA framework as an inventory-supporting auxiliary ranking component to account for practical blood availability constraints. It was used only for relative prioritization among otherwise acceptable donor units and was not intended to represent biological effects of red blood cell storage, predict storage lesions, or estimate clinical transfusion outcomes.

For A/B/O red blood cell units:

\[ Score_{storage}= \begin{cases} 10, & N\leq5\\ 12-0.4N, & N>5 \end{cases} \]

For AB red blood cell units:

\[ Score_{storage}= \begin{cases} 10, & N\leq10\\ 15-0.5N, & N>10 \end{cases} \]

The storage duration score was therefore considered an auxiliary inventory-related factor within the donor prioritization framework, whereas Rh antigen matching remained the primary decision dimension.

**Supplementary Table S6. Representative donor priority rankings under different weighting strategies**

| **Recipient Rh phenotype** | **Conservative weighting** | **Baseline AHP weighting** | **Enhanced c-antigen weighting** | **Spearman ρ** |
| --- | --- | --- | --- | --- |
| CCee | CCee > CCEe > CCEE > Ccee > CcEe > CcEE > ccee > ccEe > ccEE | CCee > CCEe > CCEE > Ccee > CcEe > CcEE > ccee > ccEe > ccEE | CCee > CCEe > CCEE > CcEe > Ccee > CcEE > ccee > ccEe > ccEE | 0.95 |
| Ccee | Ccee > CCee > ccee > CcEe > CCEe > CcEE > CCEE > ccEe > ccEE | Ccee > CCee > ccee > CcEe > CCEe > CcEE > CCEE > ccEe > ccEE | Ccee > CCee > ccee > CCEe > CcEe > CCEE > CcEE > ccEe > ccEE | 0.93 |
| ccEe | ccEe > ccee > ccEE > CcEe > CCEe > Ccee > CCee > CcEE > CCEE | ccEe > ccee > ccEE > CcEe > CCEe > Ccee > CCee > CcEE > CCEE | ccEe > ccee > ccEE > CCEe > CcEe > CCee > Ccee > CcEE > CCEE | 0.92 |
| CcEE | CcEE > CCEE > ccEE > CcEe > CCEe > Ccee > CCee > ccEe > ccee | CcEE > CCEE > ccEE > CcEe > CCEe > Ccee > CCee > ccEe > ccee | CcEE > CCEE > ccEE > CCEe > CcEe > CCee > Ccee > ccEe > ccee | 0.94 |
| Other Rh phenotypes* | See detailed ranking results | See detailed ranking results | See detailed ranking results | 0.92–0.96 |

Note: Conservative weighting reduced the relative difference between Rh antigen weights, whereas enhanced c-antigen weighting increased the relative contribution of c antigen based on its higher immunogenicity. The baseline weighting scheme was derived from the analytic hierarchy process (AHP). Donor schemes were ranked using the MCDA–TOPSIS framework. Spearman rank correlation coefficients were calculated to evaluate the consistency of ranking results between alternative weighting strategies and the baseline AHP model.

**Supplementary Table S7. Prespecified classification criteria and sensitivity analyses for MCDA-based high-match and low-match groups**

| **Classification component** | **Prespecified criterion** | **Rationale** | **Sensitivity analysis** |
| --- | --- | --- | --- |
| Rank-based classification | **High-match:** Actual transfused unit ranked No.1 among all eligible donor candidates | Rank 1 represents the highest-priority donor scheme according to the MCDA–TOPSIS framework and indicates maximal concordance with model recommendation | Patients classified using Rank 1 only as the high-match definition showed consistent direction of association |
| Near-optimal ranking | **High-match:** Rank 2 donor scheme with Rh antigen matching score ≥9 and difference from Rank 1 integrated priority score ≤10% | Rank 2 solutions with minimal score differences may represent clinically acceptable alternatives under real-world inventory constraints | Alternative thresholds (Rh antigen matching score ≥8 or score difference ≤15%) were additionally evaluated |
| Lower-priority ranking | **Low-match:** Rank ≥3 donor scheme | Lower-ranked donor schemes indicate reduced concordance with MCDA priority ordering and relatively increased antigen exposure burden | Excluding borderline Rank 3 cases did not materially alter the observed association |
| Antigen exposure criterion | **Low-match:** Rh antigen matching score ≤7 or donor carries recipient-missing RhC/c/E/e antigen | Represents donor schemes with relatively lower antigen compatibility within the MCDA decision space | Reclassification based only on Rh antigen matching score showed similar trends |
| Storage score integration | **Integrated priority score = Rh antigen matching score ×0.80 + storage duration score ×0.20** | Storage duration was incorporated as an auxiliary inventory optimization factor, whereas Rh antigen matching remained the primary decision dimension | Alternative models excluding storage duration or using different integration approaches showed similar ranking stability |

**Note:**
The classification criteria were prespecified before outcome analysis. The thresholds were determined according to the hierarchical structure of MCDA-derived donor ranking, clinical interpretability, and practical blood inventory constraints rather than empirical optimization according to outcome differences. High-match and low-match classifications reflect the concordance between actual transfusion practice and MCDA-based donor priority ranking and do not indicate clinical compatibility or incompatibility.

**Supplementary Table S8. AHP pairwise comparison matrix, derived weights, and consistency evaluation of Rh antigen weighting**

**Table S8A. Aggregated AHP pairwise comparison matrix for Rh antigen weighting**

| **Rh antigen** | **C** | **c** | **E** | **e** |
| --- | --- | --- | --- | --- |
| C | 1 | 1/9 | 1/5 | 1/3 |
| c | 9 | 1 | 3 | 5 |
| E | 5 | 1/3 | 1 | 3 |
| e | 3 | 1/5 | 1/3 | 1 |

**Note:**
The pairwise comparison matrix was constructed using the Saaty 1–9 scale. A higher value indicates that the row antigen was considered more important than the column antigen within the MCDA donor priority ranking framework.

**Table S8B. AHP-derived weights of Rh antigens**

| **Rh antigen** | **Weight** |
| --- | --- |
| c antigen | 0.5528 |
| E antigen | 0.2687 |
| e antigen | 0.1202 |
| C antigen | 0.0583 |

**Table S8C. Consistency evaluation of the AHP judgment matrix**

| **Parameter** | **Value** |
| --- | --- |
| Maximum eigenvalue (λmax) | 4.0076 |
| Consistency Index (CI) | 0.0025 |
| Random Index (RI) | 0.900 |
| Consistency Ratio (CR) | 0.0028 |

**Table S8D. Expert panel characteristics and weighting procedure**

| **Item** | **Description** |
| --- | --- |
| Number of experts | 7 |
| Transfusion medicine specialists | 5 |
| Clinical laboratory specialists | 2 |
| Expert selection criteria | ≥10 years of experience in transfusion medicine, immunohematology, or related laboratory practice |
| Evaluation basis | Published evidence regarding Rh antigen immunogenicity, regional Rh antigen distribution characteristics, and clinical transfusion practice |
| Weighting method | Analytic hierarchy process (AHP) using Saaty 1–9 scale |
| Consistency criterion | CR <0.10 considered acceptable |

Note：The AHP-derived weights represent relative decision preferences within the MCDA framework and should not be interpreted as direct estimates of biological immunogenicity.
